# Supplementary material for: Nonvesicular lipid transfer drives myelin growth in the central nervous system
Source: Nat Commun. 2024 Nov 11;15:9756. doi: 10.1038/s41467-024-53511-y (PMC11554831; doi:10.1038/s41467-024-53511-y)
Supplement: Supplementary file 1 — Supplementary Information [file 41467_2024_53511_MOESM1_ESM.pdf]

## SUPPLEMENTARY FIGURES

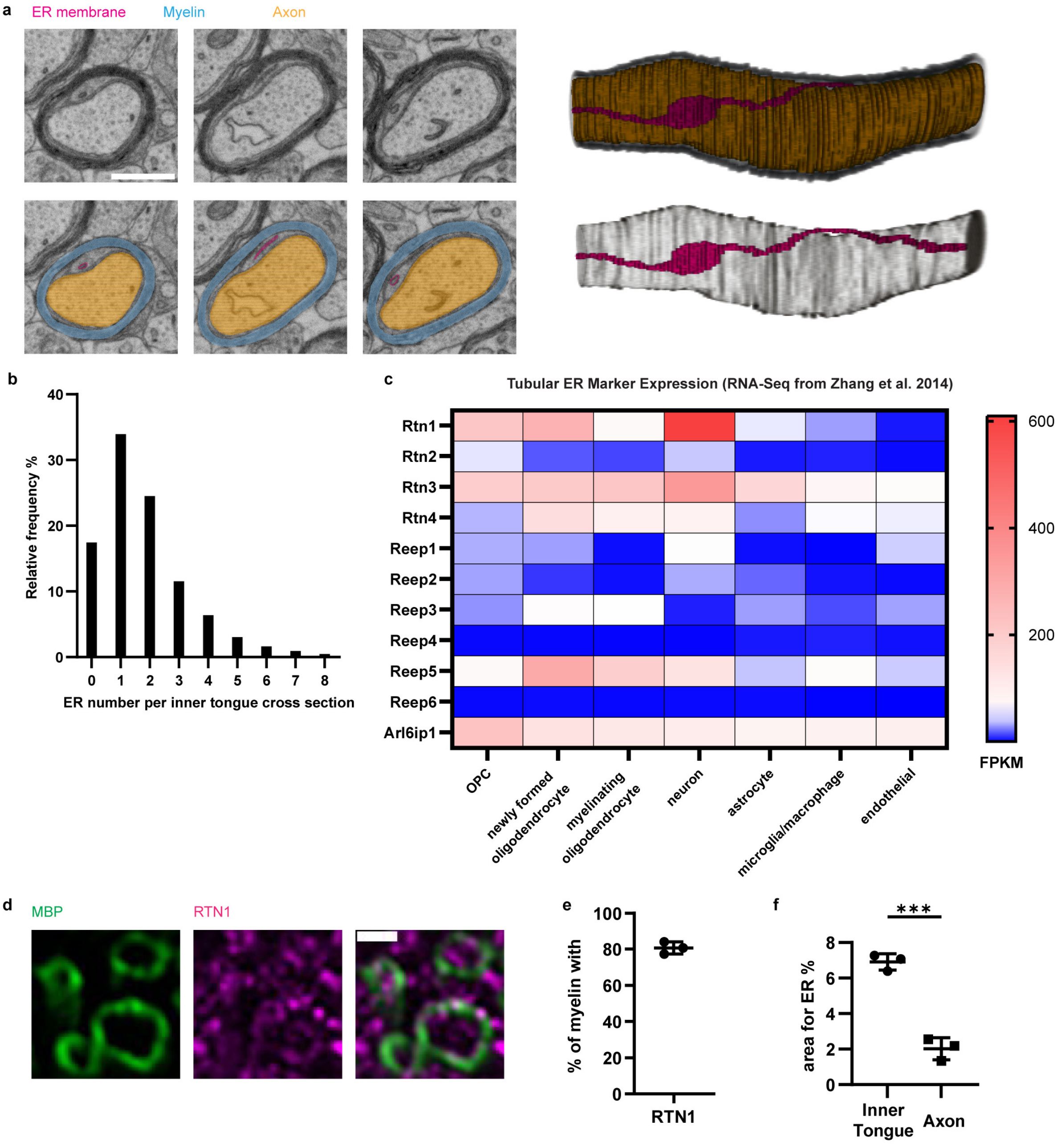


**Supplementary Fig. 1 │Tubular ER is enriched in developing myelin.**

**a** Another 3D reconstruction of ER in the inner tongue. From a 4.5µm-thick stack (91 slices with 50 nm interval). Magenta: ER membrane; Blue: Myelin; Yellow: Axon. **b** The distribution of ER number per inner tongue cross section, quantified from 425 inner tongue cross sections of three mice. **c** tubular ER marker expression profiles, analysis of RNA-Seq from Zhang et al.[41], **d** immunocytochemistry of P14 spinal cord showing tubular ER marker RTN1 puncta overlapping with MBP marked myelin. **e** 80.58 ±3.32 % (mean ± SD) of myelin overlapping with RTN1. **f** ER occupies 6.91±0.45% area of inner tongue and 2.02±0.62% area of axon (mean ± SD). (n=3 wildtype P14 mice, Two-tailed unpaired t-test, t=10.95, df=4, ***p= 0.0004) Scale bar: 0.5 µm (a) 1 µm (d). Source data are provided as a Source Data file.

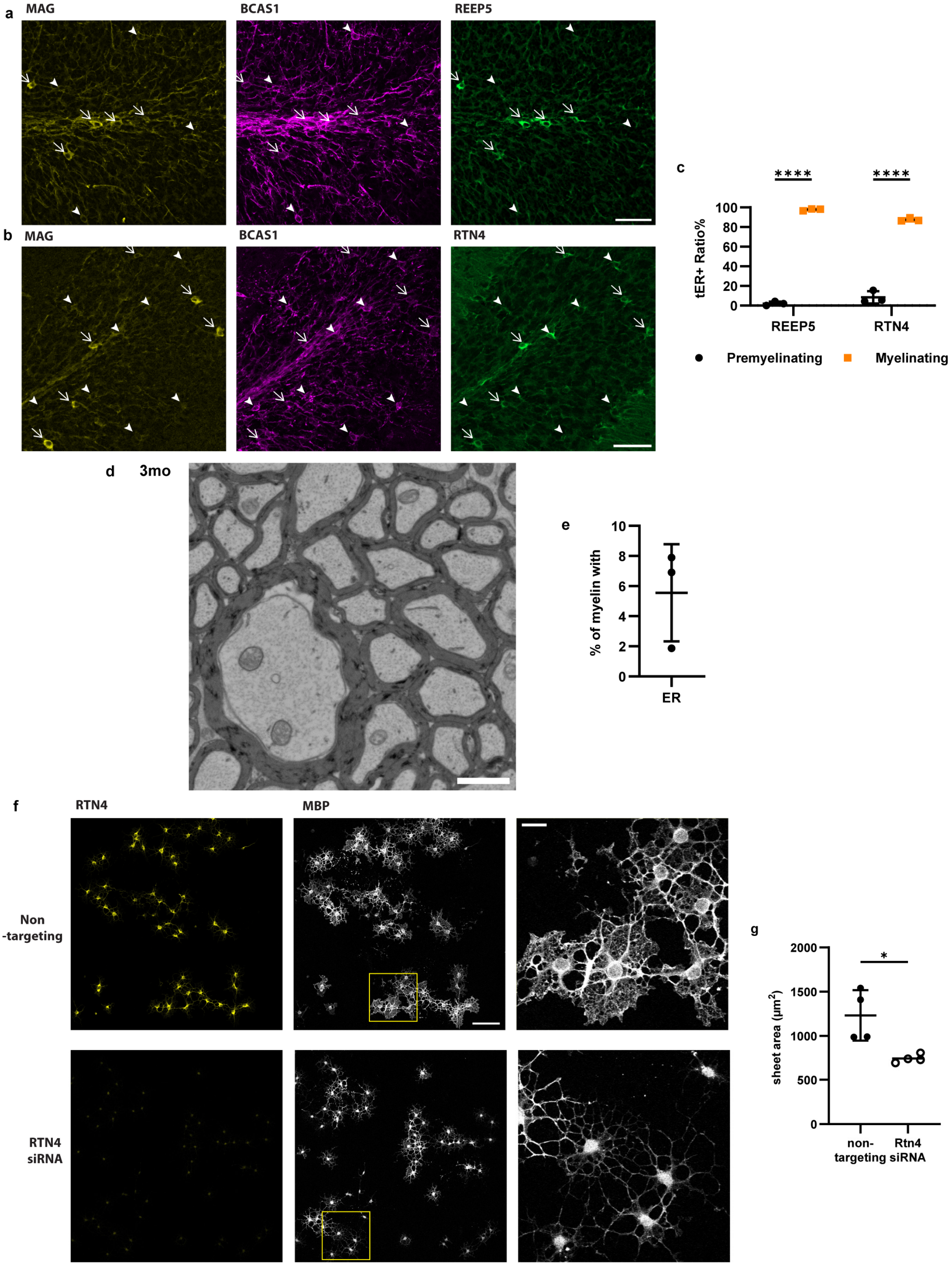


**Supplementary Fig. 2 │ Tubular ER is associated with active myelination.**

**a, b** Immunohistochemistry of P14 mouse cerebellum. Arrow heads: pre-myelinating oligodendrocytes ($MAG^{-}BCAS1^{+}$); arrows: myelinating oligodendrocytes ($MAG^{+}BCAS1^{+}$). **c** Quantification of (a) and (b) from three P14 mice, showing mean ± SD, 2.04±2.09% or 8.40±6.19% premyelinating cells are REEP5+ or RTN4+, 97.79±1.03% or 87.48±1.68% myelinating cells are REEP5+ or RTN4+. (n=3 wildtype P14 mice, Two-way ANOVA

followed by Sidak's multiple comparison test, ****p<0.0001). **d** 3-month-old (3mo) wild-type optic nerve myelin. **e** Ratio of 3mo myelin that contain the ER, showing mean ± SD: 5.55±3.23 %. **f** *Rtn4* knockdown in oligodendrocyte culture. **g** Quantification of $MBP^+$ sheet area from four independent replicates, showing mean ± SD, non-targeting siRNA: 1483±359.8, *Rtn4* siRNA: 868.1±60.03. (n=4 technical replicates, Two-tailed unpaired t-test, t=3.395, df=6, *p=0.0146) Scale bars: 50 µm (a, b), 1 µm (d), 100 µm (f), 20 µm (f zoom in). Source data are provided as a Source Data file.

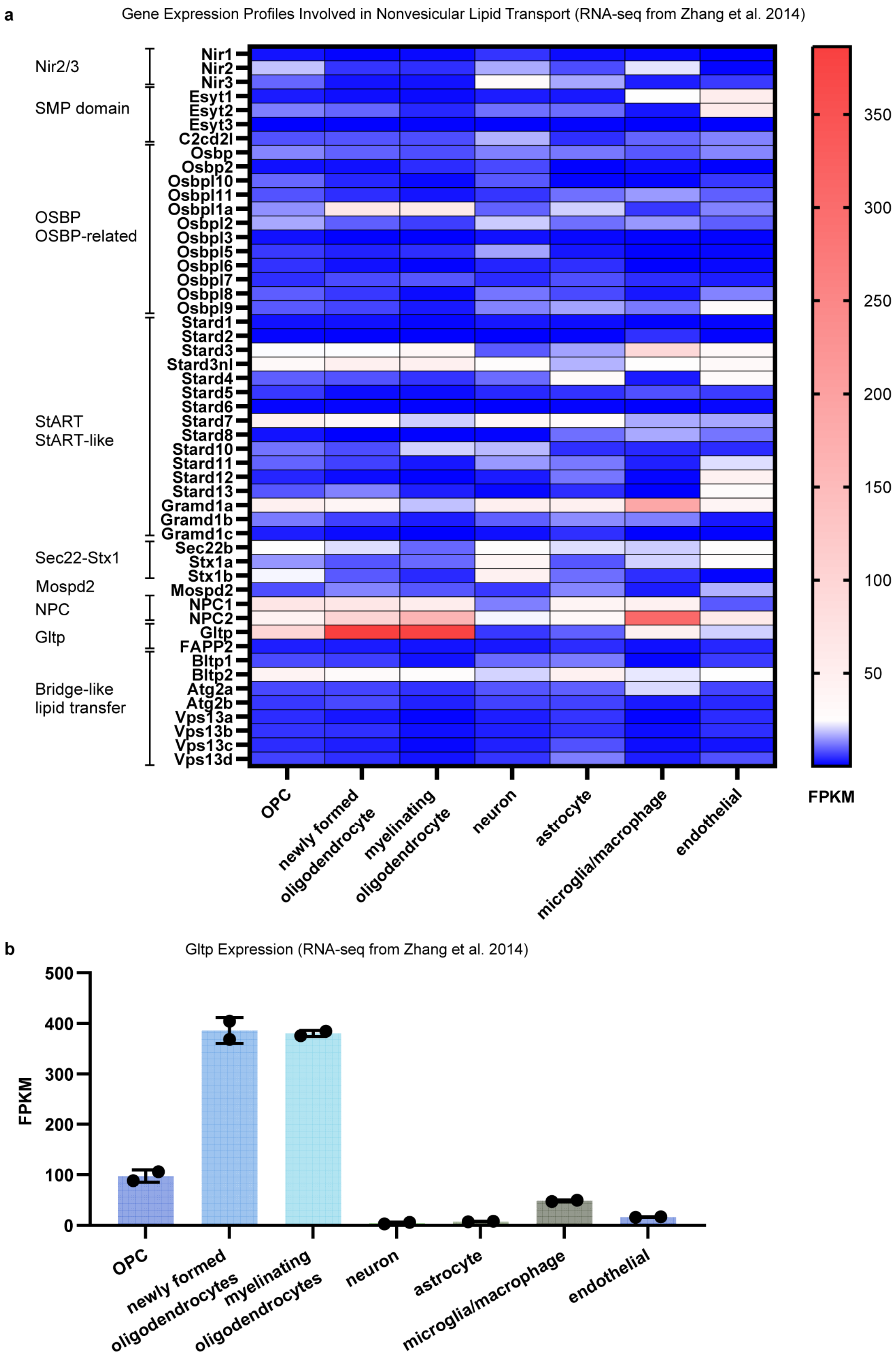


**Supplementary Fig. 3 | Glycolipid transfer protein (GLTP) is associated with active myelination**

**a** Expression profiles of genes involved in nonvesicular lipid transport, analysis of RNA-Seq from Zhang et al.[1] **b** *Gltp* expression profiles based on analysis of RNA-seq from Zhang et

al.[1] FPKM reads (two replicates per cell type) OPC: 88.495447, 105.799478; newly formed oligodendrocytes 368.317293, 404.231063; myelinating oligodendrocytes: 375.889082, 384.631206; neurons: 3.025255, 5.900611; astrocytes: 7.211993, 7.877303; microglia: 49.882491, 47.164643; endothelial cells: 15.936901, 16.717767. Source data are provided as a Source Data file.

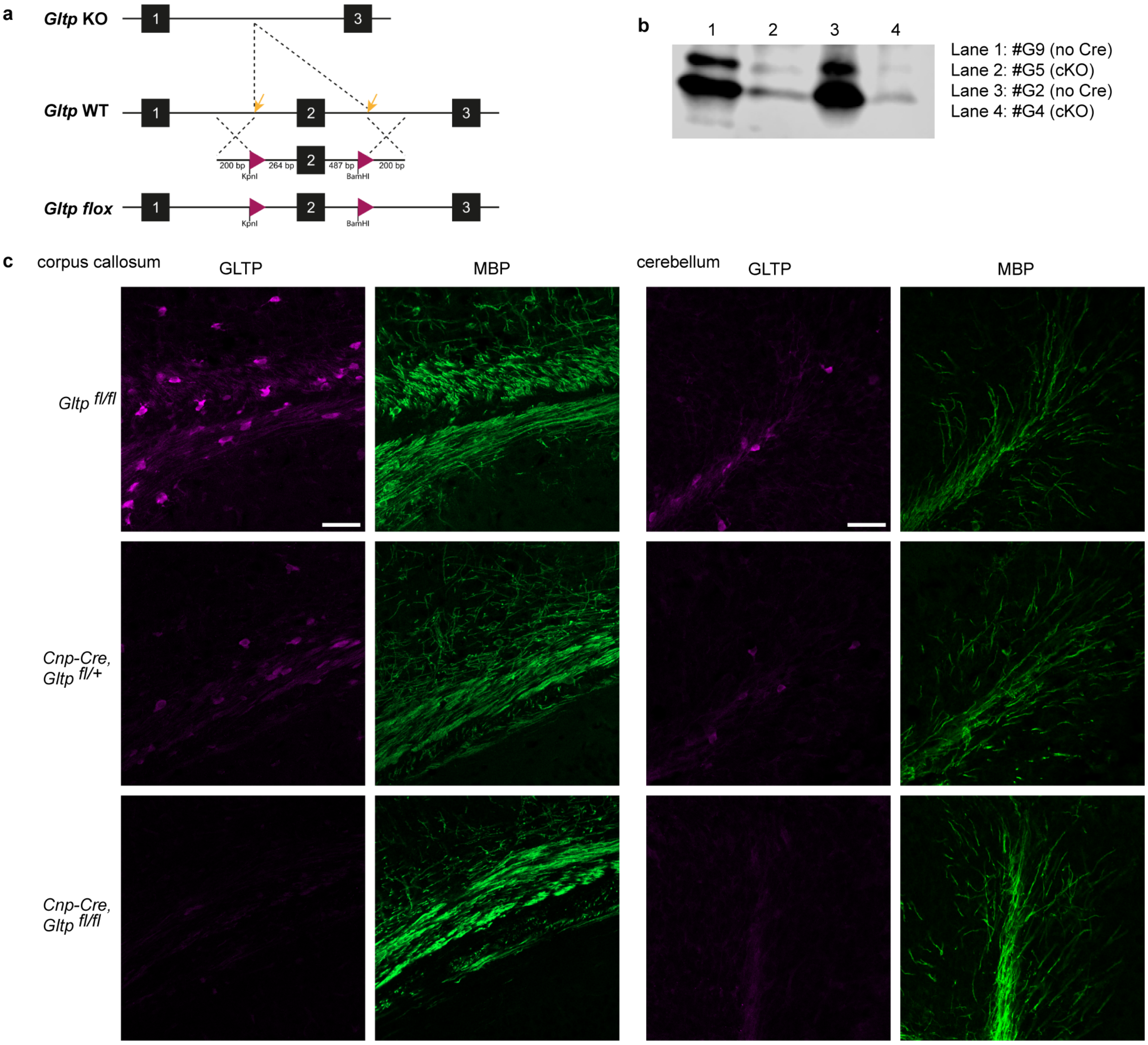


**Supplementary Fig. 4 │ Design and validations of *Gltp* mutants**

**a** Design of *Gltp* knockout (KO) or *Gltp flox* mouse lines. **b** Western Blot analysis of whole brain lysate from four P14 mice, 12 µg protein per lane. **c** Immunohistochemistry of corpus callosum and cerebellum from P14 WT (*Gltp* $^{fl/fl}$), het (*Cnp-Cre, Gltp* $^{fl/wt}$) and cKO (*Cnp-Cre, Gltp* $^{fl/fl}$). Scale bars: 50 µm (c). Source data are provided as a Source Data file.

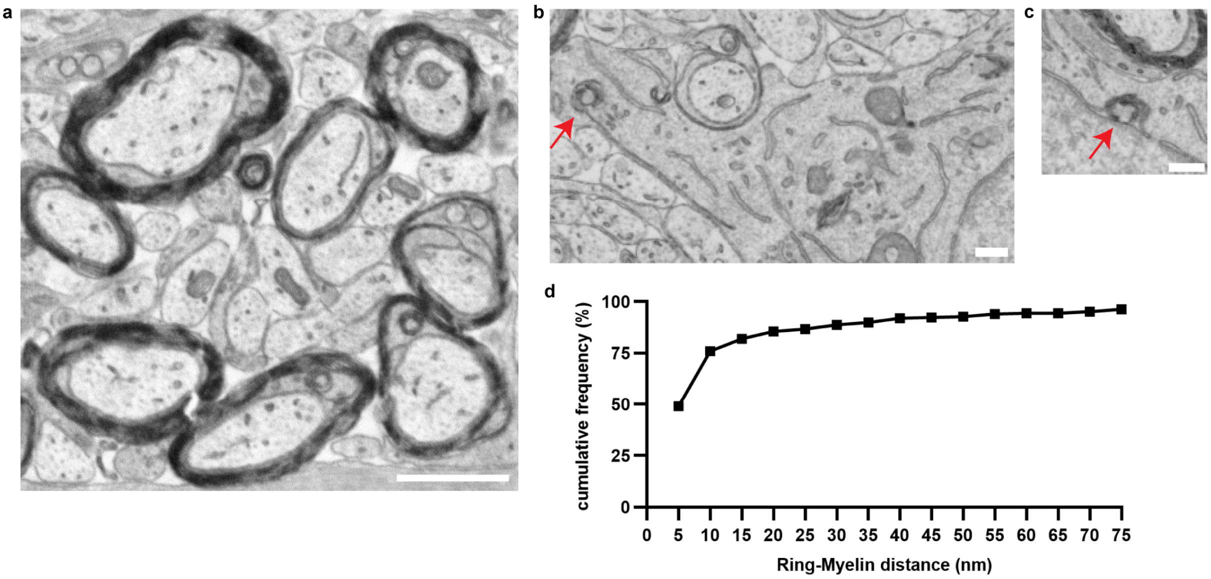


**Supplementary Fig. 5 | Mice lacking GLTP in oligodendrocytes exhibit ER pathology in myelin**

**a** Membrane rings in inner tongue at optic nerve of cKO (*Cnp-Cre, Gltp* $^{fl/fl}$). **b** Ring (red arrow) at cell body of oligodendrocyte (the corresponding 3D stack is shown in Movie 4) **c** Ring (red arrow) at nuclear envelop of oligodendrocyte. **d** Distribution of Ring-Myelin distance, analysis of 240 rings from three P14 *Gltp* cKO mouse optic nerves. Scale bars: 1 µm (a) 100 nm (b, c). Source data are provided as a Source Data file.

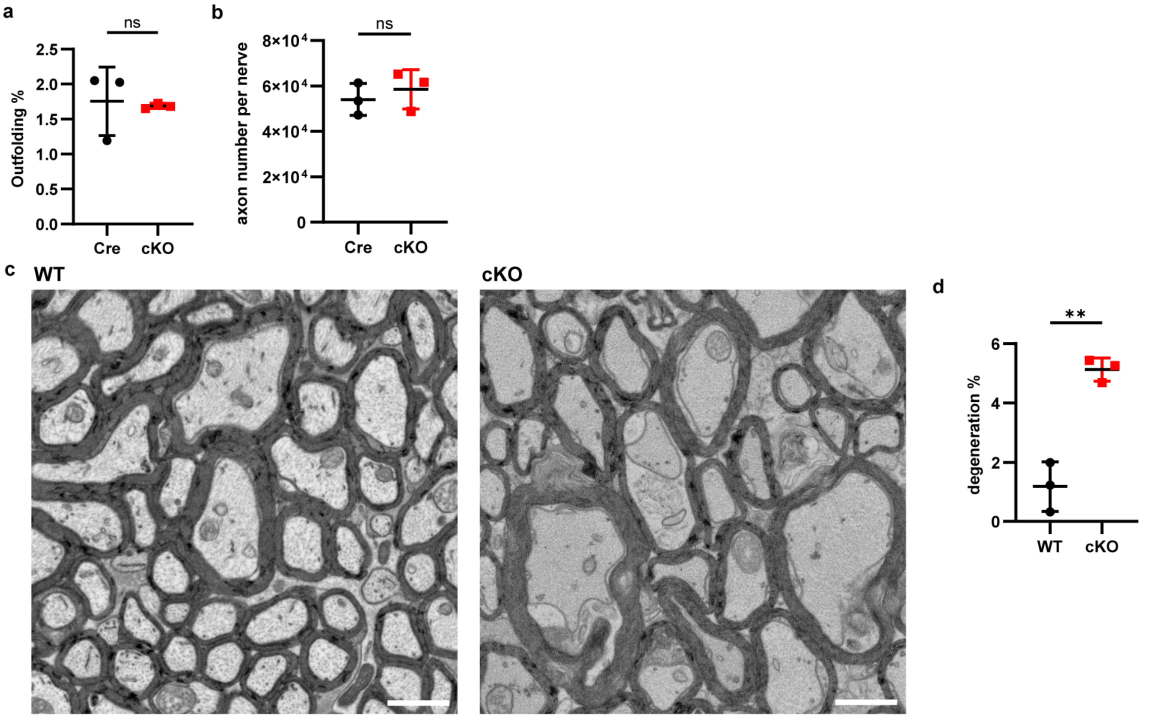


**Supplementary Fig. 6 | Effects of *Gltp* mutation at P28 and 3-month-old**

(a, b) Analysis of P28 mice: **a** Ratio of outfolding, showing mean ± SD, Cre: 1.756 ± 0.489%, cKO: 1.688 ± 0.039% (n=3 mice per condition, Two-tailed unpaired t-test, p=0.82) **b** Estimated axon number per nerve, showing mean ± SD, Cre: 54037 ± 7041, cKO: 58577 ± 8629 (n=3 mice per condition, Two-tailed unpaired t-test, p=0.52) (c, d) analysis of 3-month-old mice. **c** Representative SEM images of 3-month-old wild-type and Gltp cKO mouse optic nerve. **d** Quantification of degeneration, showing mean ± SD, WT: 1.181 ± 0.840%, cKO: 5.128 ± 0.392% (n=3 mice for each condition, Two-tailed unpaired t-test, t=7.373, df=4, **p=0.0018) Scale bar: 1 µm (c). Source data are provided as a Source Data file.

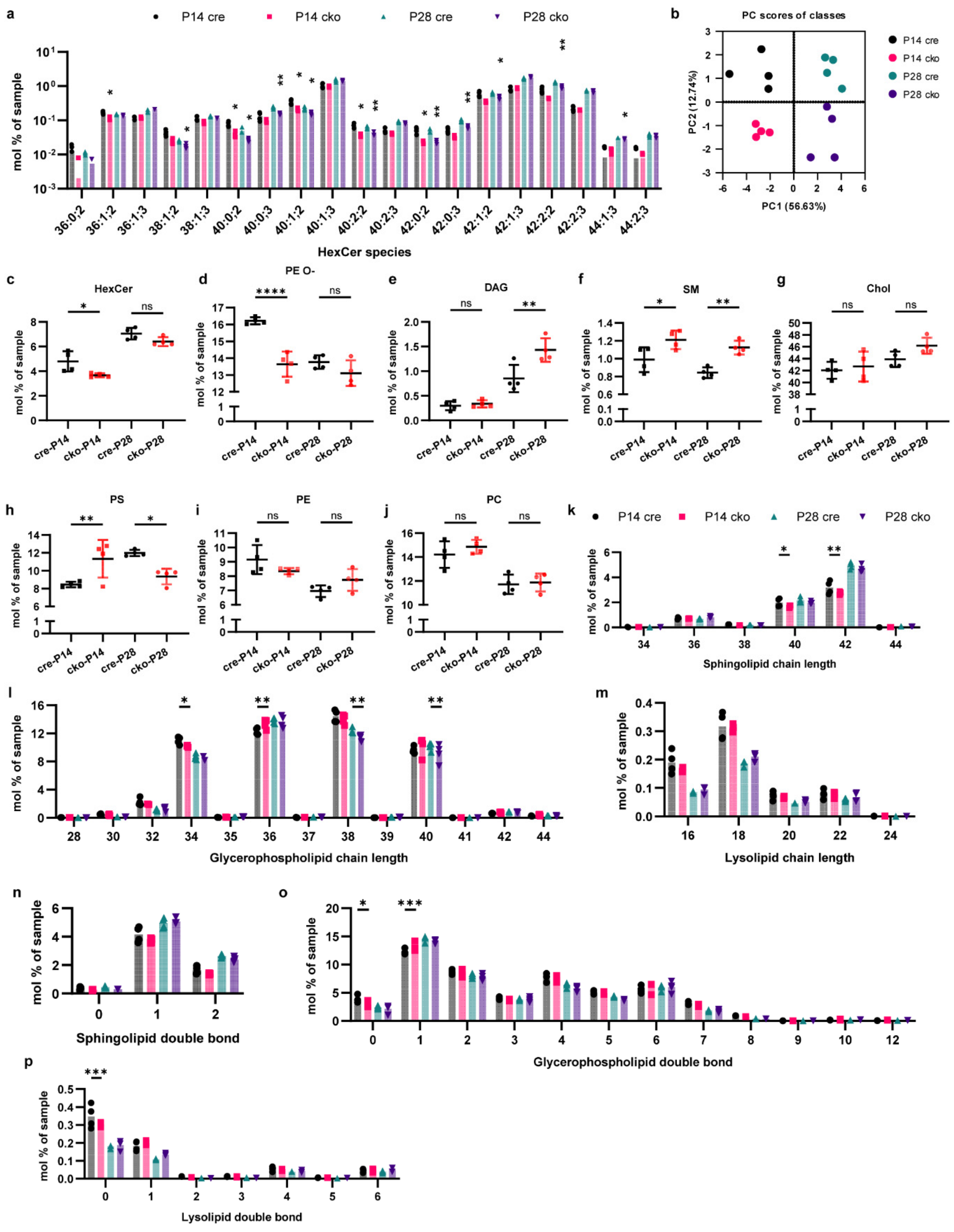

**Supplementary Fig. 7 ｜ Delivery of glycolipid to myelin is impaired in *Gltp* mutants**

**a** Relative amount of different GalCer species. P values are calculated from two-way ANOVA followed by Tukey's post-hoc test. "36:1;2 P14" *p=0.0102; "38:1;2 P28" *p=0.0292; "40:0;2 P14" *p=0.0159; "40:0;2 P28" *p=0.0449; "40:0;3 P28" **p=0.0088; "40:1;2 P14" *p=0.0436; "40:1;2 P28" *p=0.0391; "40:2;2 P14" *p=0.0277; "40:2;2 P28" **p=0.0078; "42:0;2 P14" *p=0.0322; "42:0;2 P28" **p=0.0042; "42:0;3 P28" **p=0.0074; "42:1;2 P28"

*p=0.0209; “42:2;2 P28” **p=0.0080; “44:1;3 P28” *p=0.0455 **b** Principal Component Analysis of the samples based their lipid classes. **c-j** One-way ANOVA of major lipid classes in myelin (instead of Two-way ANOVA in Fig. 6d), showing mean ± SD. P value is calculated from one-way ANOVA followed by Sidak’s multiple comparisons. “HexCer P14” *p=0.0181; “PE O- P14” ****p<0.0001; “DAG P28” **p=0.0022; “SM P14” *p=0.0163; “SM P28” **p=0.0035; “PS P14” **p=0.0087; “PS P28” *p=0.0150. **k-m** Chain length of different functional classes. P values are calculated from two-way ANOVA followed by Tukey’s post-hoc test. “Sphingolipid-40 P14” *p=0.0418; “Sphingolipid-42 P14” **p=0.0014; “Glycerophospholipid-34 P14” *p=0.0273; “Glycerophospholipid-36 P14” **p=0.0024; “Glycerophospholipid-38 P28” **p=0.0041; “Glycerophospholipid-40 P28” **p=0.0044 **n-p** Double bond of different functional classes. P values are calculated from two-way ANOVA followed by Tukey’s post-hoc test. “Glycerophospholipid-0, P14” *p= 0.0116; “Glycerophospholipid-1 P14” ***p= 0.0008; “Lysolipid-0 P14” ***p= 0.0004. Data point color coding and shapes are consistent across panels a, k–p. Source data are provided as a Source Data file.

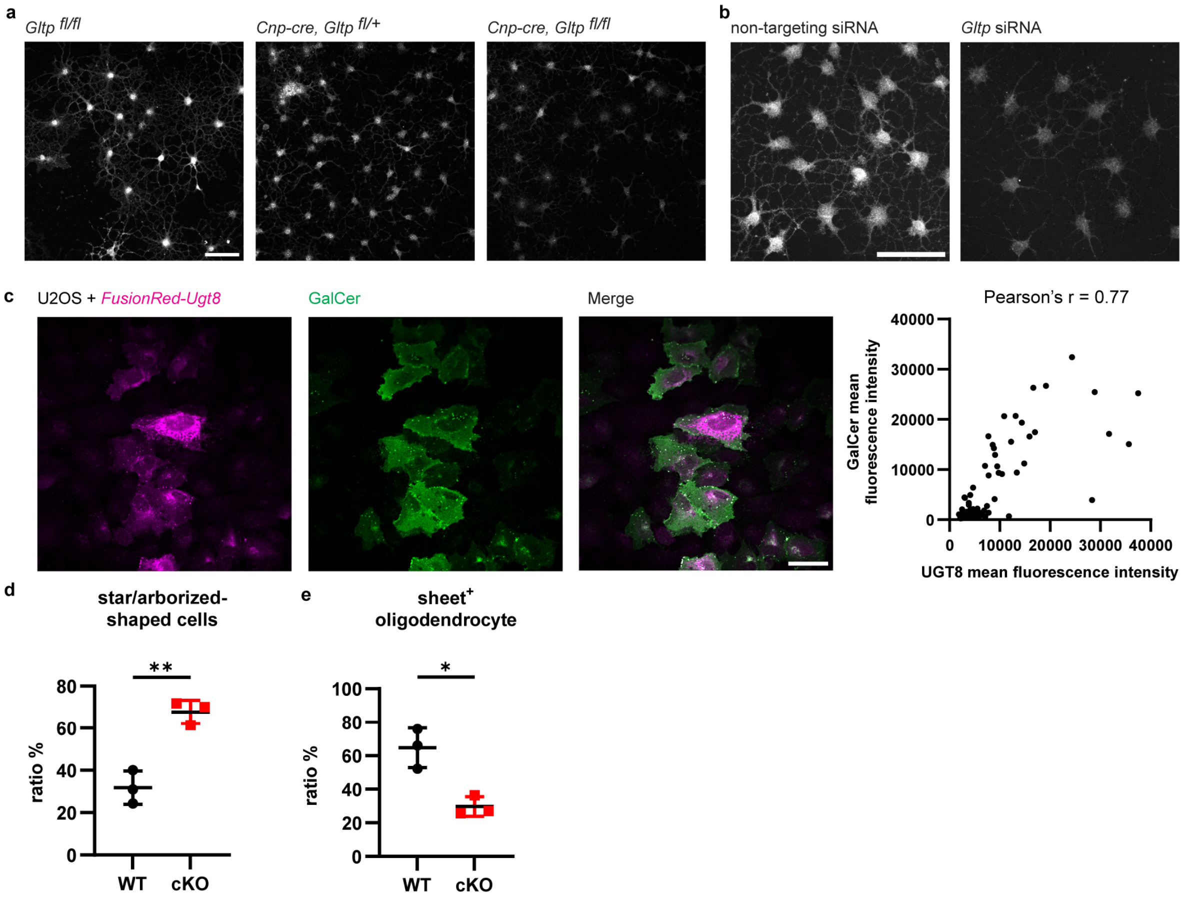


**Supplementary Fig. 8 | Supporting information for the GalCer transportation assay**

**a** Validation of *Gltp* knockout oligodendrocyte culture. **b** Validation of *Gltp* knockdown oligodendrocyte culture. **c** Validation of anti-GalCer antibody. GalCer immunofluorescence is elevated in cells overexpressing *Ugt8*, which encodes the GalCer-synthesizing enzyme UGT8. Data points represent measurement from individual cells (n=96) **d-e** Ratio of primary oligodendrocytes in different stages, showing mean ± SD, for star/arborized-shaped stage, WT: 31.76±7.89%, cKO: 67.72±5.58%, two-tailed unpaired t-test, t=6.449, df=4, **p =0.0030. For sheet$^{+}$ mature stage, WT: 64.63±11.83%, cKO: 29.67±5.84%, two-tailed unpaired t-test, t=4.590, df=4, *p= 0.0101. Scale bars: 50 µm (a, b, c). Source data are provided as a Source Data file.

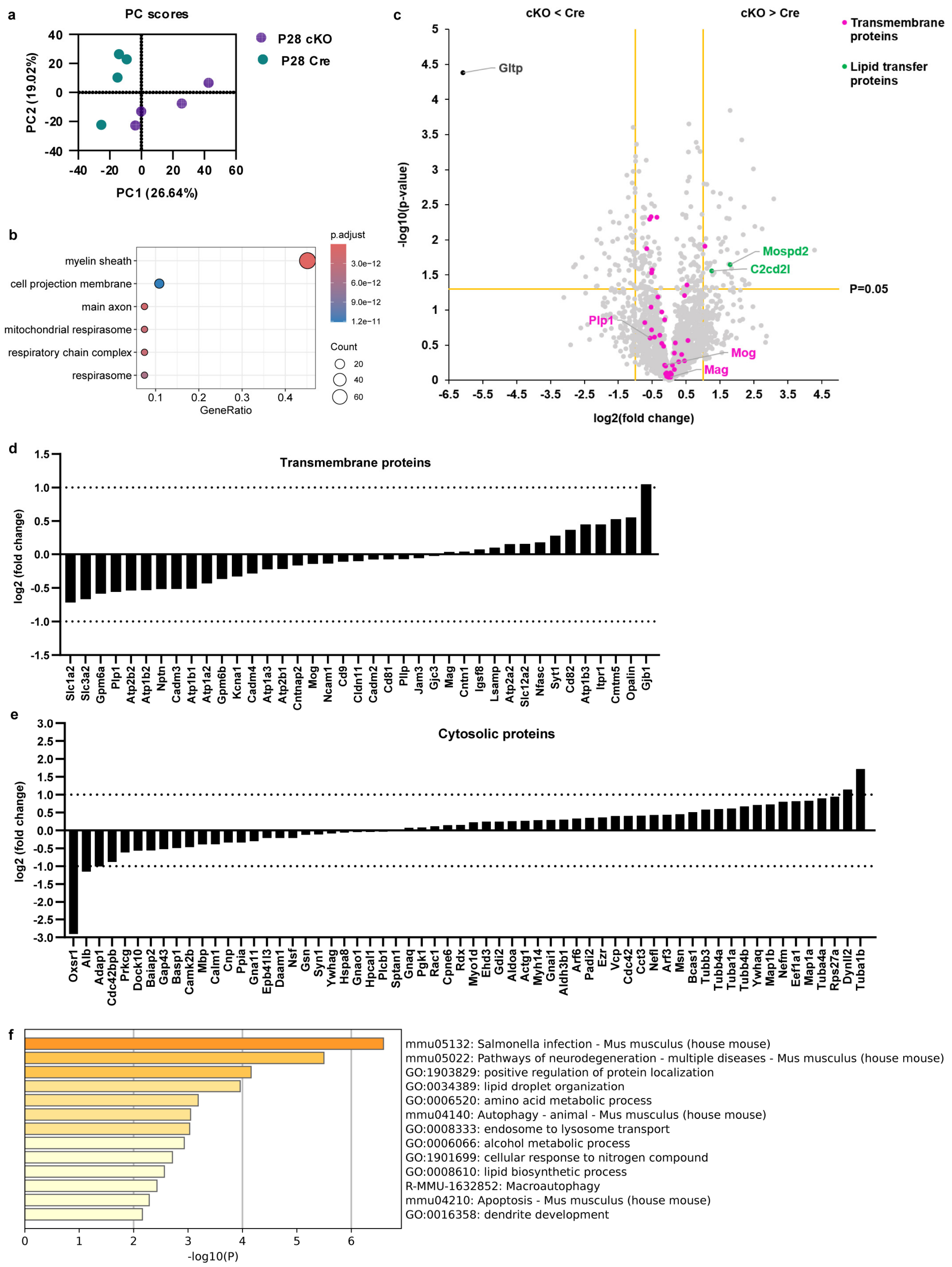


**Supplementary Fig. 9 | Myelin proteome alterations of *Gltp* cKO**

**a** Principal Component Analysis of the samples. **b** Cellular component GO term analysis showing these purified myelin samples contains mainly myelin, with some contaminations from axons and mitochondria. p.adjust are p-values adjusted using the Benjamini-Hochberg

method. **c** Volcano plot shows differentiated regulated proteins in cKO compared to. Cre control. Vertical lines mark -/+ 2 folds change. Data points above horizontal line have p-value <0.05 in two-tailed Student's t-test. Two lipid transfer proteins were upregulated in cKO: *Mospd2* and *C2cd2l*. **d** Bar plot showing fold changes of transmembrane proteins among Top 200 abundant proteins. Note that mitochondria transmembrane proteins are not included because they are known contaminant of biochemical purifications of myelin. **e** Bar plot showing fold changes of cytosolic proteins among Top 200 abundant proteins. **f** Upregulated pathways in *Gltp* cKO based on Metascape enrichment analysis [2]. Source data are provided as a Source Data file.

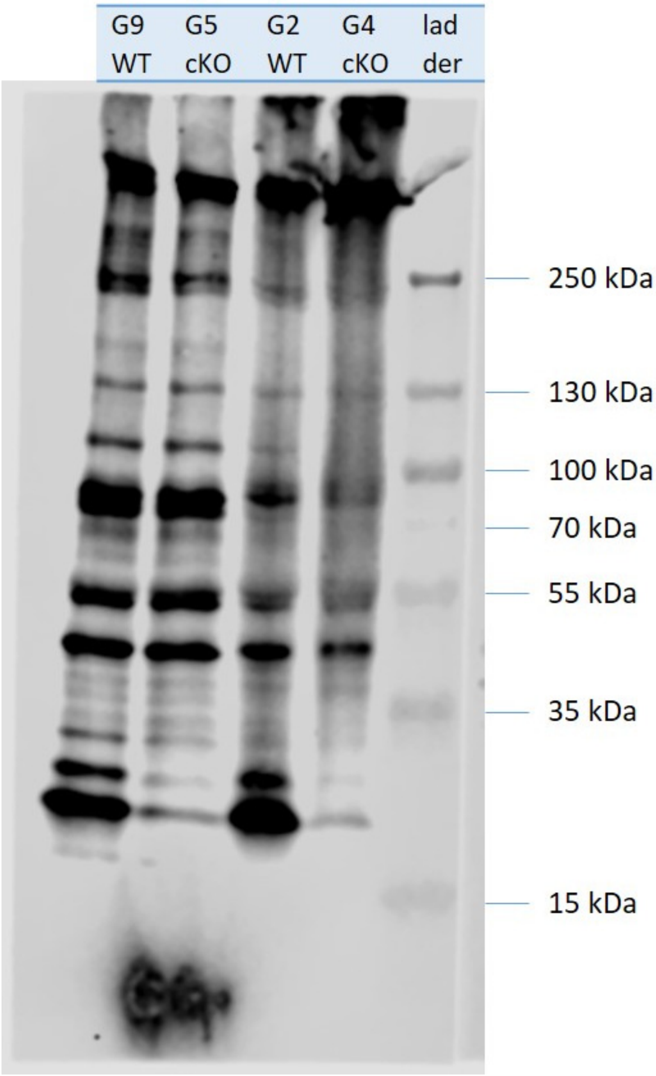


**Uncropped blot for Supplementary Fig4b**

Western Blot analysis of whole brain lysate from four P14 mice, 12 µg protein per lane, primary antibody: anti-Gltp (Sigma ATA-HPA056461-100), secondary antibody: anti-rabbit-HRP

**Reference**


1. Zhang, Y. *et al.* An RNA-sequencing transcriptome and splicing database of glia, neurons, and vascular cells of the cerebral cortex. *J. Neurosci.* **34**, 11929–11947 (2014).

2. Zhou, Y. *et al.* Metascape provides a biologist-oriented resource for the analysis of systems-level datasets. *Nat. Commun.* **10**, 1523 (2019).
