# Supplementary material for: Nonvesicular lipid transfer drives myelin growth in the central nervous system
Source: Nat Commun. 2024 Nov 11;15:9756. doi: 10.1038/s41467-024-53511-y (PMC11554831; doi:10.1038/s41467-024-53511-y)
Supplement: Supplementary file 2 — Description of Additional Supplementary Files [file 41467_2024_53511_MOESM2_ESM.pdf]

Description of Additional Supplementary Files

Supplementary Movie 1 | 3D segmentation of ER in myelin inner tongue from P14 wildtype mouse optic nerve, related to Fig 1a. Magenta: ER, Green: axon. Scale cube 1x1x1 µm.

Supplementary Movie 2 | Volume EM (ATUM-SEM) stack revealed the ring structure is a long tube (arrow), 11 µm-thick (222 slices with 50 nm interval), from P14 *Gltp* cKO mouse optic nerve. Scale bar 500nm.

Supplementary Movie 3 | Volume EM (ATUM-SEM) stack of rolling-up ring in myelin inner tongue, 1.5 µm-thick (31 slices with 50 nm interval), from P14 *Gltp* cKO mouse optic nerve. Related to Fig 4c. Scale bar 100nm.

Supplementary Movie 4 | Volume EM (ATUM-SEM) stack of rolling-up ring in oligodendrocyte cell body, 2.1 µm-thick (43 slices with 50 nm interval), from P14 Gltp cKO mouse optic nerve. Related to Supplementary Fig 5b. Scale bar 100 nm.

Supplementary Dataset 1 | Lipidomics of myelin from wild-type and *Gltp* cKO mice. Lipid species abundances (in mole %) for myelin purified from P14 Cre, P14 cKO, P28 Cre and P28 cKO. n=4 mouse for each condition.

Supplementary Dataset 2 | Proteomics of myelin from wild-type and Gltp cKO mice. (1) Top 200 hits according to the intensity. Common contaminants, reverse and site-specific identifications were excluded. Only proteins with equal or greater than 2 peptides (Peptides $\geqslant 2$) in 3 out of 4 biological replicates for each condition were quantified. (2) Significantly altered proteins with a log2 fold change above 1 in cKO compared to Cre. For the statistical analysis of cKO and WT samples, a two-sided two-sample student's t-test was utilized with a set p-value of 0.05. (3) Significantly altered proteins with a log2 fold change below -1 in cKO compared to Cre. For the statistical analysis of cKO and WT samples, a two-sided two-sample student's t-test was utilized with a set p-value of 0.05.

Supplementary Dataset 3 | Metascape enrichment analysis for upregulated pathways in Gltp cKO. The P value was calculated by one-sided accumulative hypergeometric and was used for filtering, the q value was the adjusted p value by the Benjamini-Hochberg method.
