# Supplementary material for: Nonvesicular lipid transfer drives myelin growth in the central nervous system
Source: Nat Commun. 2024 Nov 11;15:9756. doi: 10.1038/s41467-024-53511-y (PMC11554831; doi:10.1038/s41467-024-53511-y)
Supplement: Supplementary file 10 — Reporting Summary [file 41467_2024_53511_MOESM10_ESM.pdf]

nature portfolio

Corresponding author(s): Mikael Simons

Last updated by author(s): Sep 17, 2024

Reporting Summary

Nature Portfolio wishes to improve the reproducibility of the work that we publish. This form provides structure for consistency and transparency in reporting. For further information on Nature Portfolio policies, see our Editorial Policies and the Editorial Policy Checklist.

Statistics

For all statistical analyses, confirm that the following items are present in the figure legend, table legend, main text, or Methods section.

n/a	Confirmed	
☐	☒	The exact sample size (*n*) for each experimental group/condition, given as a discrete number and unit of measurement
☐	☒	A statement on whether measurements were taken from distinct samples or whether the same sample was measured repeatedly
☐	☒	The statistical test(s) used AND whether they are one- or two-sided<br>*Only common tests should be described solely by name; describe more complex techniques in the Methods section.*
☒	☐	A description of all covariates tested
☐	☒	A description of any assumptions or corrections, such as tests of normality and adjustment for multiple comparisons
☐	☒	A full description of the statistical parameters including central tendency (e.g. means) or other basic estimates (e.g. regression coefficient) AND variation (e.g. standard deviation) or associated estimates of uncertainty (e.g. confidence intervals)
☐	☒	For null hypothesis testing, the test statistic (e.g. *F*, *t*, *r*) with confidence intervals, effect sizes, degrees of freedom and *P* value noted<br>*Give P values as exact values whenever suitable.*
☒	☐	For Bayesian analysis, information on the choice of priors and Markov chain Monte Carlo settings
☒	☐	For hierarchical and complex designs, identification of the appropriate level for tests and full reporting of outcomes
☐	☒	Estimates of effect sizes (e.g. Cohen's *d*, Pearson's *r*), indicating how they were calculated

Our web collection on statistics for biologists contains articles on many of the points above.

Software and code

Policy information about availability of computer code

Data collection	(1) Fluorescence microscopy: ZEN 3.2 (Blue edition) (Zeiss);<br>(2) Scanning electron microscopy: Crossbeam Gemini 340 Atlas 5 (Zeiss);<br>(3) Transmission electron microscopy: EM-Menu software (TVIPS);<br>(4) Volume electron microscopy: Apreo S2 SEM (Thermo Fisher Scientific);<br>(5) Western blot: Image Studio TM (LI-COR).<br>(6) Lipidomics and Proteomics: Q Exactive (Thermo Scientific)
Data analysis	(1) Fluorescence microscopy: Fiji;<br>(2) Electron microscopy: Fiji, VAST, Blender, MyelTracer;<br>(3) Statistical Analysis: Microsoft Excel 2016, GraphPad Prism 10. Volcano Plot (Fig. 6a) additionally used Lipid Map statistical tool (https://github.com/metabolomicsworkbench/jupyter-notebooks/blob/master/MWPerformVolcanoPlotAnalysis.ipynb);<br>(4) Lipidomics: LipidXplorer (Lipotype)<br>(5) Proteomics: Perseus (version 2.0.7.0)

For manuscripts utilizing custom algorithms or software that are central to the research but not yet described in published literature, software must be made available to editors and reviewers. We strongly encourage code deposition in a community repository (e.g. GitHub). See the Nature Portfolio guidelines for submitting code & software for further information.

Data

Policy information about availability of data

All manuscripts must include a data availability statement. This statement should provide the following information, where applicable:

- Accession codes, unique identifiers, or web links for publicly available datasets
- A description of any restrictions on data availability
- For clinical datasets or third party data, please ensure that the statement adheres to our policy

Data supporting the findings of this manuscript are available from the corresponding authors upon reasonable request. Source data are provided with this paper.

Research involving human participants, their data, or biological material

Policy information about studies with human participants or human data. See also policy information about sex, gender (identity/presentation), and sexual orientation and race, ethnicity and racism.

Reporting on sex and gender	n/a
Reporting on race, ethnicity, or other socially relevant groupings	n/a
Population characteristics	n/a
Recruitment	n/a
Ethics oversight	n/a

Note that full information on the approval of the study protocol must also be provided in the manuscript.

Field-specific reporting

Please select the one below that is the best fit for your research. If you are not sure, read the appropriate sections before making your selection.

- [x] Life sciences
- [ ] Behavioural & social sciences
- [ ] Ecological, evolutionary & environmental sciences

For a reference copy of the document with all sections, see nature.com/documents/nr-reporting-summary-flat.pdf

Life sciences study design

All studies must disclose on these points even when the disclosure is negative.

Sample size	No power analyses were used to predetermine sample sizes. However, sample sizes were chosen based on prior literature using similar experimental paradigms.
Data exclusions	No data were excluded from analysis.
Replication	For all mouse experiments, three to four mice per genotype were analyzed. For immunohistochemistry, two to four sections per animal were analyzed. For cell culture experiments, three to four replicates per condition were analyzed.
Randomization	The processing order of samples in any given experiment is random. For cell culture experiments, wells were randomly assigned to different siRNA or transfection. For electron microscopy, four to six random fields of views from each animal were analyzed.
Blinding	Data acquisition and analysis were performed in a blinded manner when possible. The immunohistochemistry comparison between pups and adults (eg. Fig 2d,f) is not blinded due to the size difference of sections but the analysis is automated.

Reporting for specific materials, systems and methods

We require information from authors about some types of materials, experimental systems and methods used in many studies. Here, indicate whether each material, system or method listed is relevant to your study. If you are not sure if a list item applies to your research, read the appropriate section before selecting a response.

Materials & experimental systems

n/a	Involved in the study
☐	☒ Antibodies
☐	☒ Eukaryotic cell lines
☒	☐ Palaeontology and archaeology
☐	☒ Animals and other organisms
☒	☐ Clinical data
☒	☐ Dual use research of concern
☒	☐ Plants

Methods

n/a	Involved in the study
☒	☐ ChIP-seq
☒	☐ Flow cytometry
☒	☐ MRI-based neuroimaging

Antibodies

Antibodies used	Primary Antibodies:<br>1. rat anti-MBP (Abcam ab7349, 1:500 for IHC/ICC)<br>2. chicken anti-MBP (Thermo PA1-10008, 1:500 for IHC/ICC)<br>3. anti-RTN4 (Abcam ab47085, 1:500 for IHC/ICC)<br>4. anti-REEP5 (Proteintech 14643-1-AP, 1:500 for IHC/ICC)<br>5. anti-BCAS1 (Santa Cruz, sc136342, 1:400 for IHC)<br>6. anti-MAG-Alexa647 (Santa Cruz sc-166849 AF647, 1:100 for IHC 1 hour room temperature)<br>7. anti-RTN1 (Sigma HPA044249, 1:250 for ICC)<br>8. anti-SEC61B (Sigma HPA049407, 1:400 for ICC)<br>9. anti-KDEL (Enzo ADI-SPA-827-D, 1:250 ICC)<br>10. anti-GLTP (Sigma ATA-HPA056461-100, 1:300 for IHC/ICC with antigen retrieval, 1:500 for Western Blot)<br>11. anti-GalCer (Merck MAB342, 1:1500 for ICC),<br>12. anti-GalCer-Alexa488 (Merck MAB342A4, 1:100 for ICC)<br>13. anti-UGT8 (Proteintech 17982-1-AP, 1:500 for ICC)<br>14. anti-CD140a (Biolegend 135902, 1:300 for primary culture isolation)<br><br>Alexa Fluor 488–, 647–, and 555–conjugated antibodies (Invitrogen) were used 1:1000 as secondary antibodies for IHC/ICC. Goat anti-Rat IgG (Jackson ImmunoResearch 112-005-167) was used 1:333 for primary culture isolation.
Validation	All primary antibodies were validated in this study or previously validated for the species and application (see citations provided on manufacturers websites for the above catalogue numbers).

Eukaryotic cell lines

Policy information about cell lines and Sex and Gender in Research

Cell line source(s)	Primary culture of oligodendrocyte precursor cells were isolated from mice of indicated genotype, using an immunopanning protocol (PMID: 29093197). U2OS cell line was from ATCC #HTB-96.
Authentication	Primary oligodendrocytes were positive for oligodendrocyte marker MBP in immunocytochemistry.
Mycoplasma contamination	U2OS cells were tested negative for Mycoplasma.
Commonly misidentified lines<br>(See ICLAC register)	No commonly misidentified lines were used in this study.

Animals and other research organisms

Policy information about studies involving animals; ARRIVE guidelines recommended for reporting animal research, and Sex and Gender in Research

Laboratory animals	Mouse: C57BL/6J, age P7-P9, P14, 6 months<br>Mouse: Cnp-cre x Gltp flox, age P7-P9, P14, P28<br>All mice used in this study were on C57BL/6J genetic background.
Wild animals	This study did not involve wild animals.
Reporting on sex	Both male and female mice were utilized but sex-matched among cohorts for comparison.
Field-collected samples	This study did not involve samples collected from the field.
Ethics oversight	All animal experiments performed in this work were in agreement with the German animal welfare law and state specific regulations

for animal experiments.

Note that full information on the approval of the study protocol must also be provided in the manuscript.

Plants

Seed stocks	*Report on the source of all seed stocks or other plant material used. If applicable, state the seed stock centre and catalogue number. If plant specimens were collected from the field, describe the collection location, date and sampling procedures.*
Novel plant genotypes	*Describe the methods by which all novel plant genotypes were produced. This includes those generated by transgenic approaches, gene editing, chemical/radiation-based mutagenesis and hybridization. For transgenic lines, describe the transformation method, the number of independent lines analyzed and the generation upon which experiments were performed. For gene-edited lines, describe the editor used, the endogenous sequence targeted for editing, the targeting guide RNA sequence (if applicable) and how the editor was applied.*
Authentication	*Describe any authentication procedures for each seed stock used or novel genotype generated. Describe any experiments used to assess the effect of a mutation and, where applicable, how potential secondary effects (e.g. second site T-DNA insertions, mosiacism, off-target gene editing) were examined.*
